# Specific situations may require different relative indications

**DOI:** 10.1002/ccr3.3744

**Published:** 2021-01-08

**Authors:** Tamer Mohamed Zaalouk, Zouheir Ibrahim Bitar, Ossama Sajeh Maadarani

**Affiliations:** ^1^ Critical Care Unit Ahmadi Hospital Kuwait Oil Company Fahaheel Kuwait

**Keywords:** anticoagulation, dilated cardiomyopathy

## Abstract

In specific situations such as patient with severely dilated left ventricle (LV) and spontaneous echo contrast (SEC) who suffered an ischemic stroke previously may be an acceptable indication for oral anticoagulation to prevent further TE events.

## Graphical abstract

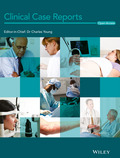

## 1 CASE PRESENTATION

Heart failure (HF) associated with endocardial and endothelial dysfunction in addition to intracardiac stasis which facilitates formation of thrombus. HF is the second most common cause of cardioembolic stroke after atrial fibrillation.^1^ Although trials in HF patients in sinus rhythm have failed to demonstrate a net clinical benefit of oral anticoagulation to prevent thromboembolic (TE) events,^2^ in specific situations such as patient with severely dilated left ventricle (LV) and spontaneous echo contrast (SEC) who suffered an ischemic stroke previously may be a relatively acceptable indication for oral anticoagulation to prevent further TE events in this specific situation.

A 48‐year‐old gentleman admitted with an exacerbation of chronic heart failure. He is a known case of ischemic cardiomyopathy and severely depressed systolic function. He had ischemic stroke 2 months prior to admission treated with thrombolytic therapy. Echocardiography demonstrated severely dilated hypokinetic LV with SEC inside LV (normal gain in echo setting) (^[Link]^, ^[Link]^). Based on history of stroke and echo findings in young patient with relatively low bleeding risk, we replaced antiplatelet with oral anticoagulation in order to prevent further TE events in such specific situation.

## CONFLICT OF INTEREST

Not declared.

## AUTHOR CONTRIBUTIONS

TZ and OM: collected the information and drafted the manuscript. ZB: revised and approved the final manuscript. Our working Web site is www.kockw.com Kuwait Oil company, Ahamdi hospital.

## CONSENT

Informed consent was obtained from the patient for the publication of this clinical image.

## Supporting information

Video S1: Parasternal long axis view

Video S2: Parasternal short axis view
